# Considerations for designing chemical screening strategies in plant biology

**DOI:** 10.3389/fpls.2015.00131

**Published:** 2015-04-08

**Authors:** Mario Serrano, Erich Kombrink, Christian Meesters

**Affiliations:** ^1^Plant Biology, Department of Biology, University of FribourgFribourg, Switzerland; ^2^Chemical Biology Laboratory, Max Planck Institute for Plant Breeding ResearchKöln, Germany; ^3^Department of Chemical Biology, Faculty of Biology, Center for Medical Biotechnology, University of Duisburg-EssenEssen, Germany

**Keywords:** *Arabidopsis thaliana*, bioactive small molecules, chemical genetics, chemical libraries, high-throughput screening, structure–activity relationship, target identification

## Abstract

Traditionally, biologists regularly used classical genetic approaches to characterize and dissect plant processes. However, this strategy is often impaired by redundancy, lethality or pleiotropy of gene functions, which prevent the isolation of viable mutants. The chemical genetic approach has been recognized as an alternative experimental strategy, which has the potential to circumvent these problems. It relies on the capacity of small molecules to modify biological processes by specific binding to protein target(s), thereby conditionally modifying protein function(s), which phenotypically resemble mutation(s) of the encoding gene(s). A successful chemical screening campaign comprises three equally important elements: (1) a reliable, robust, and quantitative bioassay, which allows to distinguish between potent and less potent compounds, (2) a rigorous validation process for candidate compounds to establish their selectivity, and (3) an experimental strategy for elucidating a compound's mode of action and molecular target. In this review we will discuss details of this general strategy and additional aspects that deserve consideration in order to take full advantage of the power provided by the chemical approach to plant biology. In addition, we will highlight some success stories of recent chemical screenings in plant systems, which may serve as teaching examples for the implementation of future chemical biology projects.

## Introduction

Forward genetic screenings have been widely used to identify the genetic elements behind biological traits. The isolation of mutants with particular phenotypes from a randomly mutagenized population is an unbiased process with the obvious advantage of targeting genes without prior knowledge of their functions. Traditionally, the identification of the responsible gene by mapping *via* experimental crosses was the most tedious and time-consuming step in this process. The advent of next-generation sequencing greatly facilitated this process, allowing genetic mapping and gene identification in relatively short time (Prioul et al., 1997; Miki and Mchugh, 2004; Schneeberger et al., 2009; Austin et al., 2011; Nordström et al., 2013). However, forward genetic screening approaches will reach their limits under three unfavorable circumstances: (1) when multiple genes are responsible for one single trait (i.e., redundancy of gene function), (2) when a gene product is crucial for survival of an organism (i.e., lethality due to loss of gene function), or (3) when a single gene is responsible for multiple phenotypes (i.e., pleiotropy of gene function).

It has been suggested and eventually demonstrated that these limitations can be circumvented by chemical genetic approaches (Schreiber, 1998; Stockwell, 2000; Blackwell and Zhao, 2003). This method relies on small bioactive molecules that modulate protein function, either by acting as agonist or antagonist thereby mimicking modification of the encoding gene products. In case of redundancy of gene function, the advantage is that a chemical compound (e.g., inhibitor) may target several proteins with identical or similar function (e.g., isoenzymes) if corresponding ligand binding sites are present. Such chemicals can be applied to plants with different genetic backgrounds or to different plant species to phenocopy genetic mutations (e.g., creating chemical instead of genetic knock-outs). Correspondingly, in cases of mutant lethality, application of a chemical (e.g., inhibitor) may be delayed to developmental stages, when the corresponding gene function is no longer essential. Since chemicals can be applied not only at different stages, but also at different concentrations, dosage-dependent phenotypes could be created, and the chemical phenotype could even be reversed (i.e., back to wild type) if a soluble compound is washed out again, thereby extending the experimental repertoire for circumventing mutant lethality.

Already characterized compounds are well-accepted as chemical tool, such as the phosphoinositide 3-kinase inhibitor wortmannin, the inhibitor of vesicular transport brefeldin A, the bacterial phytotoxin coronatine or variations of the protease inhibitor E-64 (Murphy et al., 2005; Samaj et al., 2006; Kolodziejek and Van Der Hoorn, 2010; Wasternack and Kombrink, 2010). Of course, many more such selective compounds exist. For example herbicides, which usually target primary metabolic processes that are necessary for growth and development of plants, played fundamental roles in understanding aspects of plant processes, such as photosynthesis, cell wall physiology or function of microtubules (Dayan et al., 2010). However, by using already existing chemical tools, plant biologists depend on discoveries from pharmacological screenings (Grozinger et al., 2001; Zhao et al., 2003) or random findings and are limited in case no chemical tool is available for a particular research area. Therefore, the challenge is to find novel compounds by using plant systems for chemical screening to expand the repertoire of chemical tools that target a large diversity of biological functions (Walsh, 2007; Hicks and Raikhel, 2012; Dayan and Duke, 2014).

**Figure 1:**
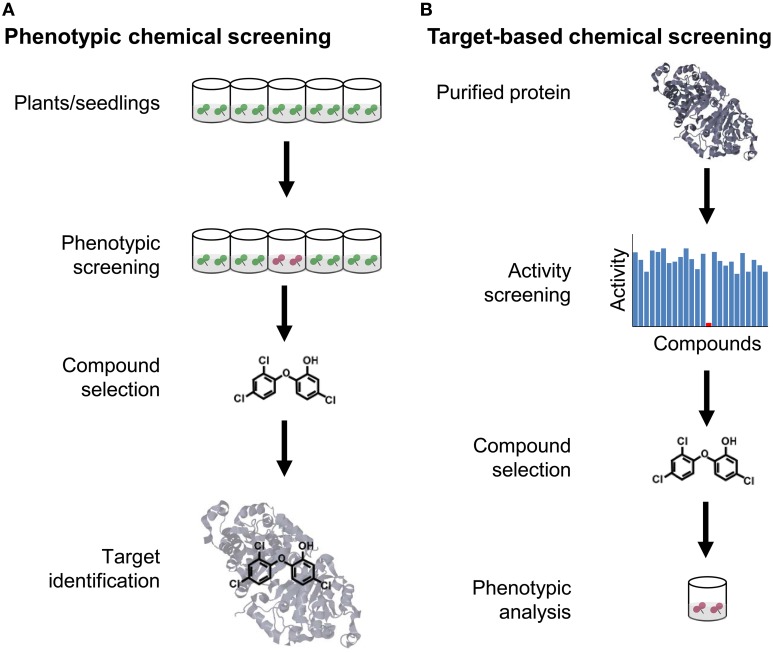
**Comparison of forward and reverse chemical screening. (A)** The goal of phenotypic or forward chemical screening is to identify from an arrayed library of chemicals a (selective) bioactive compound causing a phenotypic alteration, usually in a microplate format. Once a selective compound is found, the molecular target is identified, either by a genetic approach or some type of biochemical purification strategy. **(B)** The goal of target-based or reverse chemical screening is to identify a compound that modulates the activity of a selected protein. Subsequently, the chemical is used to determine the phenotypic consequences when applied to plants.

Similar to genetic screenings, which can be carried out in forward and reverse direction, one can distinguish between forward and reverse screening strategies in chemical genetics (Figure 1). Commonly, phenotypic or forward screening approaches aim at dissecting a biological process in animal or plant systems *via* identification of novel bioactive small molecules that selectively modulate any of the molecular components contributing to the phenotype. This approach aims at similar components as forward genetics and is unbiased with respect to the chemical's target and thus well-suited for basic research (Hicks and Raikhel, 2012). By contrast, a target-based or reverse screening approach aims at identifying chemicals that selectively interfere with a defined target. This strategy is often applied in pharmaceutical research when novel agonists or antagonists of drug targets that have been recognized as important are wanted. Such screening can be based on any protein-mediated phenotype such as enzymatic activity, protein-protein interactions or transcription factor binding (Subramaniam et al., 2001; Jung et al., 2005; Zabotina et al., 2008). The importance of target-based screenings in pharmaceutical research is reflected by the fact that half of the experimental and marketed drugs target only five protein families: G protein-coupled receptors, protein kinases, proteases, nuclear receptors, and ion channels (Inglese et al., 2007). Such limitation to few targets seems reasonable for applied research, but less suited for basic research, because it does not allow exploration of new phenotypes and new areas of biology with chemical tools (Eggert, 2013).

For the purpose of this review, we primarily use the term “chemical biology” to refer to the overall strategy of identifying and applying chemical tools for dissecting biological systems, whereas “chemical genetics” more specifically refers to combinations of chemicals with genetic approaches. In our view, a chemical biology approach comprises the following three essential elements: (1) a robust, reliable and quantitative readout to screen for small bioactive molecules, (2) a rigorous validation process to characterize selected candidate compounds, and (3) a strategy for target identification, which can be dismissed in the target-based approach. However, these three components are not sufficient for chemical screening projects, since additional elements and details need to be considered. In the following first part of this review, we will outline and discuss the general strategy of chemical biology projects, thereby providing guidelines for designing successful screenings, for hit selection and validation, and for identification of targets and modes of action. In the second part, we will describe selected examples of chemical biology projects in plant biology to highlight some characteristics of success stories of plant chemical screenings.

## Strategy to identify chemical tools

**Figure 2:**
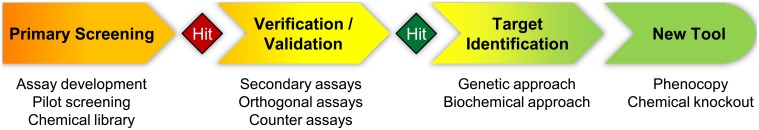
**Workflow of chemical biology screening strategy**.

When conventional genetic methods fail to answer a biological question, a chemical biology approach should be considered. It is clear that not each genetic project can easily be adapted to a chemical biology approach, because this requires different resources, experimental methodology and experience. This may be one of the reasons, why the potential of plant chemical biology has not yet been fully exploited, despite the fact that plants are attractive and well-suited for such an alternative approach. For example, the model plant *Arabidopsis thaliana* is small and can easily be grown in microplates. With its flexible culture conditions and the abundance of mutants, including a large number of reporter lines expressing diverse marker genes, it allows for dissection of virtually every signaling pathway or biological response provided it can be analyzed at the seedling stage (Hicks and Raikhel, 2012). Alternatively, cultured cells derived from *Arabidopsis* or other non-model plants are likewise amenable for facile chemical manipulation in microplates. Thus, there are ample opportunities for applying chemical screens and an enormous potential for new discoveries in the plant sciences. The general strategy to identify new chemical tools is fairly simple and in the end little specialized equipment is required, such as a versatile microplate reader. However, particular attention should be paid to the screening methodology, which includes careful design and critical assessment of the bioassay used for the primary screening, careful planning of subsequent secondary assays for validation of selected hit compounds and principle considerations concerning target identification strategies (Figure 2). Thus, it is important to see the primary screening only as the first step in a composite process leading to the development and application of new chemical tools.

### The design of a chemical screening campaign

Entering a chemical screening campaign requires enduring commitment and appropriate resources (e.g., chemical library, multimode microplate reader, or other monitoring device). Thus, careful strategic planning will help to avoid pitfalls and maximize useful outputs.

#### Assay development

An important step before starting a chemical screening is to invest into assay development. It is imperative that screening is based on a reliable, reproducible, and robust bioassay. First, it needs to be considered, whether the phenotype is suitable for scoring in the microplate format, which is inevitable for the screening process, in particular when large numbers of chemicals are involved and cumulating in high-throughput screening (HTS). For target-based approaches, such as *in vitro* enzyme assays, it is easy to use microplates with 384 or 1536 wells, but for growing single seedlings, plates with at maximum 96 wells are required. However, single-plant measurements may compromise reproducibility and in order to increase the confidence and robustness of the readout it may be beneficial to grow multiple plants in larger wells (48- or 24-well plates). In general, any phenotype that can be recorded in the microplate format is suitable for chemical screenings. However, an important consideration is to design assay conditions that allow acquisition of quantitative data during the screening, preferably in an automated fashion. Clearly, quantitative screening data will allow the application of statistical procedures and automatic, unbiased hit selection by setting threshold values. Furthermore, quantitative screening data permit to distinguish compounds with strong or weak activities, which may be useful to have for identification of new bioactive chemical scaffolds. Among quantitative readouts, fluorescence provides very strong signal intensity and is therefore the most widely used detection method in HTS in the animal field, allowing direct visualization in the tissue (Fan and Wood, 2007). In contrast, the signal strength of luminescence is significantly lower compared to fluorescence, but it exhibits an enormous dynamic range, which is mainly due to almost complete absence of background signal. Therefore, bioluminescence is an emerging method in HTS (Fan and Wood, 2007).

Acquisition of quantitative data in plant chemical biology can easily be achieved by automatic multimode microplate readers capable of recording luminescence, fluorescence and/or absorbance as generated by reporters such as luciferases, β-glucuronidase (GUS) or fluorescent proteins (Stewart, 2001; Ruijter et al., 2003). In addition, numerous biosensors exist that allow detection and quantification of intracellular concentrations of particular small molecules, including calcium ions (using aequorin), phosphate (using rhodamin-labeled phosphate binding protein), nitric oxide (using the fluorescent indicator 4,5-diaminofluorescein-2 diacetate (DAF-2DA)) and many others (Okumoto et al., 2012). For selection of an appropriate reporter system it is obvious that interference with fluorescence of chlorophyll, cell walls, and other cellular components should be avoided (Ruijter et al., 2003). Despite the obvious advantages, only few chemical screenings in plant science were based on quantitative data that were collected from diverse systems such as cultured cells, isolated membrane fractions, excised maize coleoptiles, or *Arabidopsis* seedlings analyzing absorption after quantitative staining, radioactivity of radiolabeled UDP-glucose, plant extracts *via* HPLC, or luminescence of a luciferase reporter line (Zabotina et al., 2008; Nishimura et al., 2012; Noutoshi et al., 2012; Tóth et al., 2012; Meesters et al., 2014). Remarkably, most chemical screenings with microplate-grown seedlings have assessed visible phenotypes, which can only be scored with less ease and reliability (see Supplementary Table 1 for a list of plant chemical screenings). These phenotypes include inhibition of germination, growth expansion of tissues (e.g., roots, hypocotyls), bleaching of seedlings, accumulation of secondary products (e.g., flavonoids), changes in gravitropic response or chromogenic staining using the GUS reporter. Automated image-based screenings using enhanced microscopy methods and image processing software to record phenotypes at a cellular level will be good options to enable quantification of such phenotypes and to extend the phenotypes available to HTS in plant sciences (Hicks and Raikhel, 2009). *Arabidopsis* is by far the most frequently employed plant and only few screenings have used alternative systems such as cultured tobacco cells, *in vitro* germination and growth of pollen tubes or non-plant systems such as yeast (Zouhar et al., 2010; Yoneda et al., 2007; Robert et al., 2008; Drakakaki et al., 2011; Noutoshi et al., 2012) (cf. Supplementary Table 1).

Finally, the reliability and robustness of the assay for screening purposes needs to be validated. Therefore, it is crucial to test both positive and negative controls in order to assess the dynamic range and signal variation for the experimental setup and to determine the reproducibility. The actual screening should also include both controls; thereby it can be estimated, whether candidate hits can be identified with a high degree of confidence. Acquisition of quantitative data also allows statistical analysis as determination of the screening window coefficient, called *Z*′ factor, which is a common quality metric for evaluation and validation of HTS assays, reflecting signal dynamic range, and the data variation (Zhang, 1999). The *Z*′ factor is defined in terms of four parameters: the means of both the positive (μ_pc_) and negative controls (μ_nc_) and their respective standard deviations (σ_pc_, σ_nc_) (see Formula 1).

(1) $$Z^{\prime }\text{ factor}=1-\frac{\left(3\sigma_{pc}+3\sigma_{nc}\right)}{\left|\mu_{pc}-\mu_{nc}\right|}$$

**Figure 3:**
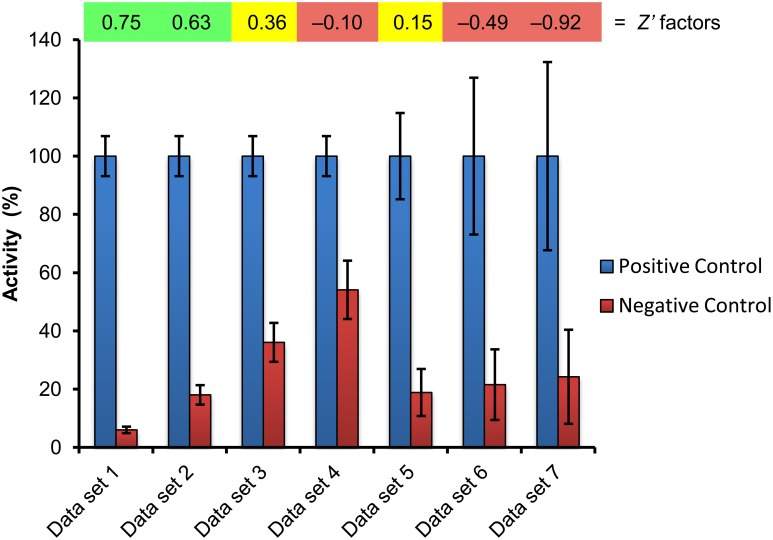
**Estimation of assay quality by *Z*′ factor determination**. The positive and negative controls included with a screening plate (cf. Figure 4) were used to calculate the *Z*′ factor, which is shown above the corresponding data set. The values of data set 1 are from a real experiment recently published (Halder and Kombrink, 2015), and the resulting *Z*′ factor of 0.75 indicates that this is an excellent assay for screening (quantification of GUS activity). Gradual, hypothetical increase of the negative control value (data sets 2–4) reduces the screening window and correspondingly the *Z*′ factor, leading to marginal (*Z*′ = 0.36) and unacceptable (*Z*′ = −0.10) assay quality for screening purposes. Likewise, increasing variability of assay data leads to decreasing *Z*′ factors and assay quality (data sets 5–7). Green, yellow, red indicate excellent, marginal, and inacceptable assay quality, respectively.

The *Z*′ factor ranges from negative infinity to 1, and a high value (>0.5) defines an excellent assay, a low value (>0) an acceptable assay and a negative value (<0) an ineffective assay with too much overlap between the positive and negative controls for the assay to be useful (Figure 3). However, it is fair to mention that this stringent statistical parameter was developed to evaluate and validate HTS assays, and although in principle useful, it may be too rigorous for application to bioassays in complex plant systems such as whole seedlings, which are prone to variability. Particular care is required when the assay requires scoring of a qualitative phenotype. Under these circumstances, measures have to be installed that generate reliable and comparable data sets from which hits can be extracted with high confidence. This may include direct application of a second readout during the screening process or subjecting only selected positive hits to a useful alternative bioassay as previously demonstrated for a number of screening campaigns (Gendron et al., 2008; De Rybel et al., 2009; Forde et al., 2013; Hu et al., 2014).

#### Hit selection

**Figure 4:**
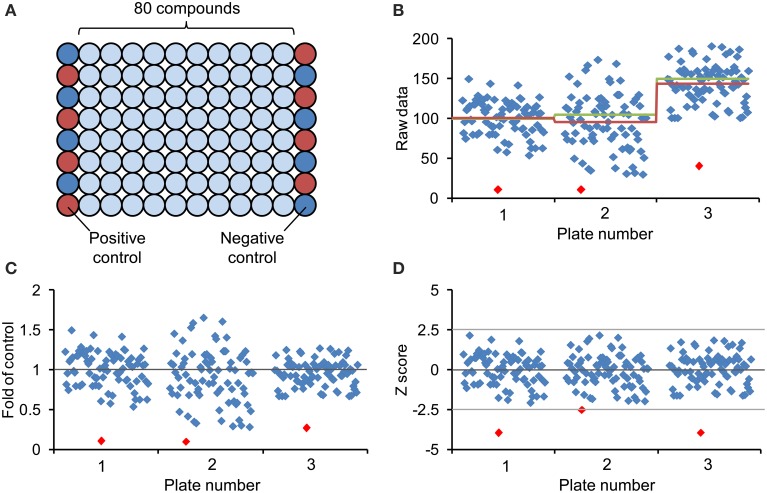
**Design of chemical screening plate and methods for data normalization and visualization. (A)** Generally, commercial arrayed chemical libraries are provided with 80 different compounds stored in the middle of 96-well plates and the first and last columns are left empty. Correspondingly, column 1 and column 12 are available for controls and to minimize edge-related bias, the eight positive controls (red circles) and the eight negative controls (blue circles) are distributed across these columns in alternating order. **(B)** Scatter plot of hypothetical screening data showing three different plates. The red line represents the corresponding plate average, the green line the mean of assumed control value (included on each of the plates), which are not necessarily identical with the plate average. **(C)** Representation of screening data after normalization to plate average (fold of control). **(D)** Screening data after normalization by *Z* score transformation (see text). The *Z* score dampens the plate-to-plate variation and increases confidence in hit selection by introduction of a common threshold value. Notice that the lowest value of each plate (red diamond) may not fulfill the cut-off criteria after *Z* score transformation.

Chemical screenings can be performed in different ways, with single or replicate measurements. The use of replicates allows a minimum of statistical analysis and thereby gives improved confidence in hit selection by reducing the number of false positive or negative hits. With small chemical libraries (<500 compounds) it is feasible and convenient to screen with replicates, but when large chemical libraries (>2000 compounds) are used, it is worthwhile to consider time, labor and costs, as these will proportionally increase. Therefore, current practice in drug discovery is to omit replicates (such screenings may involve >100,000 compounds), which requires very robust and reliable bioassays (Malo et al., 2006). However, for non-commercial screening projects in plant science, with libraries rarely exceeding 10,000 compounds (Supplementary Table 1) the advantages of replicate measurements prevail the drawbacks. In conjunction with replicate measurements, two additional points deserve consideration: (1) The library size should be related to the number of expected (and finally uncovered) hit compounds, as these will subsequently require thorough characterization. The expected hit rate increases with the target space (number of potential targets) and will be higher with readouts that are dependent on a large network (e.g., hormone signaling), whereas the target space is limited in case of short signal transduction chains comprising only few components. It is difficult to put numbers on the expected hit rate because, irrespective of theoretical considerations, it will largely depend on the stringency of hit selection. Based on our own experience using quantitative and qualitative screenings, hit rates vary between less than one and up to few percent (Serrano et al., 2007, 2010; Meesters et al., 2014). (2) The question of how to design the microplate setup should be answered for any library screening. As mentioned earlier, it is useful to include control treatments on each plate. The problem of potential plate-to-plate variation should not be underestimated, especially when a screening campaign extends over longer time periods, and appropriate controls help to normalize and better compare quantitative readouts and to identify outliers and deviating plates. Because of possible positional effects, the controls should ideally be randomly distributed across the plate, which is of course not very convenient. However, chemical libraries are often delivered in microplates with the first and last columns left empty, which can be used for respective controls. An efficient way for arranging the controls is to use alternate wells for positive and negative controls along these two columns (Figure 4) (Malo et al., 2006).

Selection of hits in qualitative screenings can result in subjective and arbitrary decisions. Such bias can be avoided by selecting hits on the basis of quantitative, normalized data. Many different methods have been developed to normalize quantitative data (for review see Malo et al., 2006). Common normalization approaches include “factor or percent of control” (FOC, POC) and “factor or percent of sample,” which are easy to calculate and interpret (Figure 4). However, the first method requires a large number of controls to provide an adequate estimation of their mean, whereas the latter method omits controls altogether and instead relates each sample values to the mean of all samples on the plate, which is a valid assumption provided that most compounds on a plate are inactive and thus can serve as controls. Similarly, the classical *Z* score or *Z* transformation—not to be confused with the *Z*′ factor mentioned above—also excludes control measurements but incorporates the sample variation and relating it to within-plate variation of all samples. Specifically, the Z score is calculated by subtracting from each sample value (*x_i_*) the mean of all plate values (*x*) and dividing this difference by the standard deviation of all measurements (σ_*x*_) (see Formula 2).

(2) $$Z\;score=\frac{x_{i}-\bar{x}}{\sigma_{x}}$$

All normalization procedures described above can only account for systematic plate-to-plate variation but not for within-plate systematic effects, such as extreme edge or row effects or other indicators of technical problems. To cope with these, the *B* score and other statistical methods are available that make minimum assumption about positional effects and may be applied to remove systematic row, column or well-effects. However, since these calculations are based on an iterative algorithm and since complex biological systems such as plant seedlings provide rather variable data, it is not easy and may not be appropriate or possible to estimate the *B* score. Based on these considerations, we recommend applying the *Z* score to chemical screening data in plant systems. Following *Z* transformation of the raw data, the mean of all measurements is represented by zero (0) in the plate-well scatter plot (Figure 4). The highly variable values from a hypothetical screen of three 96-well plates cover the range from +2.5 to -2.5 standard deviations around the mean and by defining an appropriate threshold value (e.g., −2.5 standard deviation), the *Z* score allows objective selection of hit compounds (Figure 4D). Of course, the *Z* score can also be based on control values rather than the plate average (provided it is based on sufficient data). In fact, such added controls may serve to verify the assumption for using the plate average and may also help to identify unexpected problems such as an unusual high number of positive hits. Since the *Z* score, when based on the arithmetic mean, is sensitive to statistical outliners, the substitution of the mean and standard deviation by the outlier-insensitive median and median absolute deviation results in a robust *Z* score.

Eventually, the identification of “hits” or “screening positives” is the goal of any screening campaign and it is essential to subject only the most promising compounds to the subsequent work flow (Figure 2). Thus, hit selection is the critical process of deciding which sample values differ meaningfully from the controls. For screens based on qualitative data, the selection might be biased by the wish to not miss potentially valuable hits. Therefore, phenotypic screening may be prone to high rates of false positive hits. It may be useful to develop a rating system for phenotypical strength or to select only a limited percentage of compounds showing the highest scores. By contrast, quantitative screening data are less prone to biased hit selection, but of course, the hit rate is affected by the setting of the corresponding threshold value. Such variable adjustment will allow subjecting as many compounds as feasible and convenient to confirmatory re-screening. Of note, less stringent selection criteria will increase the number of false positive hits and correspondingly reduce the number of false negative hits (Malo et al., 2006).

Finally, to ensure a successful screening, data acquisition and analysis should go hand in hand, i.e., data should be analyzed while the screen is in progress, to allow the identification of problems as they occur. It is important to visualize the raw data as well as the transformed and normalized values because these might indicate different technical problems (Figure 4) (Birmingham et al., 2009). Alternatively, it is advisable to perform a pilot screening using a small number of selected molecules with defined bioactivity or a small chemical library (see next section). Such pilot screening gives an impression about the variability of the assay under screening conditions and may indicate the expected hit rate, which should be considered in selecting the size of a chemical library.

### Chemical libraries and screening concentration

A collection of small molecules—commonly referred to as chemical library—is the starting point for performing the actual chemical screening. Ideally, the compounds of such library should have general properties that allow for high selective bioactivity, such as low molecular weight, the capacity to pass through membranes and strong and effective interaction with their targets (Smukste and Stockwell, 2005). The bioavailability of a chemical in a biological system depends on its solubility, uptake, distribution and metabolism within the organism. In pharmaceutical drug research, the Lipinski's rule of five (RO5) describes molecular properties for orally administered human drugs that would make it likely to be taken up into cells (Lipinski et al., 1997). The parameters include the molecular mass (<500 dalton), the octanol-water partition coefficient (log *P* < 5), number of hydrogen bond donors (N-H and O-H bonds <5) and hydrogen acceptors (N and O atoms <10). Note that all numbers are multiples of five, which is the origin of the rule's name. However, the rule does not predict if a compound is pharmacologically active, it rather describes physicochemical properties that from experience are favorable for drugs and, correspondingly, violation of at least one of these criteria generally makes a compound less suitable as a drug. As a rule of thumb, there are many exceptions to Lipinski's rule. For example, it was shown that sets of herbicides and insecticides do not comply with the RO5 (Tice, 2001, 2002), indicating that bioavailability of small molecules may significantly differ between organisms or their particular mode of action. Therefore, not only compounds in compliance with RO5 may be of interest, in particular when screening in plant systems.

The success of a chemical screening campaign is intimately connected not only with the assay and screening design, but also with the selection of the appropriate chemical library. Numerous chemical libraries are commercially available, which differ in size, composition and chemical diversity. Since these collections are usually designed for drug research, they mostly comprise RO5 compliant compounds (Shelat and Guy, 2007). In industrial research settings, HTS of very large libraries (>100,000 compounds) is facilitated by automation, using one or more robots for sample handling and data collection. By contrast, in initial screenings in plant systems that were carried out in academic environments, less than 100 molecules were analyzed (Min et al., 1999; Hayashi et al., 2001). More recently, plant chemical screening projects have also successfully employed more than 40,000 compounds (see Supplementary Table 1). However, when considering library size, it has to be balanced with screening effort and cost as well as with the expected hit rate. It needs to be critically assessed, how many candidate compounds identified in a primary screening can eventually be carried through all subsequent characterization and selection steps. There is a number of examples that valuable compounds were identified from relatively small compound collections (Min et al., 1999; Hayashi et al., 2001; Serrano et al., 2007; He et al., 2011; Tóth et al., 2012; Meesters et al., 2014).

Chemical libraries not only differ in size, but also in composition and the nature of compounds, which may affect the screening strategy and the outcome of a screening project as discussed in detail (for drug screening) elsewhere (Shelat and Guy, 2007). Here it suffices to briefly describe five relevant categories of libraries to provide a basis for general considerations: (1) Bioactive collections (libraries of bioactive compounds) contain compounds with well-characterized biological activities (e.g., protein kinase inhibitors). Such libraries (usually smaller in size) are useful because they facilitate narrowing down or even identifying molecular targets. (2) Natural product libraries are assembled from compounds isolated from various organisms. They are considered to provide higher hit rates, because they comprise compounds that are synthesized and transported in biological systems and might therefore bind to related protein scaffolds in a heterologous system (Koehn and Carter, 2005; Li and Vederas, 2009). (3) RO5 libraries represent the majority of screening collections. They are typically derived from chemical synthesis and may suffer from limited structural diversity when containing multiple derivatives of certain templates. (4) To enhance the structural complexity of chemical libraries, diversity-oriented synthesis (DOS), and biology-oriented synthesis (BIOS) strategies have been developed, aiming at novel chemotypes with high complexities that resemble natural products (Schreiber, 2000; Shelat and Guy, 2007; Kaiser et al., 2008). (5) Fragment libraries represent another special case of compound collections that is being used for certain screening strategies that aim at identifying only substructures (fragments) of bioactive molecules that are subsequently optimized by chemical modification (Carr et al., 2005). Relevant for plant screening projects have so far been only compound collections of categories 1 through 3 and combinations thereof.

A special collection of bioactive compounds of interest for the plant research community is the Library of AcTive Compounds on Arabidopsis (LATCA) that Sean Cutler and colleagues assembled from diverse chemical libraries, such as LOPAC (Sigma-Aldrich), and Spectrum (Microsource) and other screening collections (Chembridge, Maybridge), as well as common inhibitors, herbicides, plant hormones and research chemicals (http://cutlerlab.blogspot.de/2008/05/latca.html; accessed December 2014). The selection was based on activity in various phenotypic screens of *Arabidopsis* seedlings monitoring hypocotyl length (Zhao et al., 2007). Thus, this collection of about 3600 compounds with proven activity in plant systems is a good starting point for screening projects and hits can potentially be associated with known pathways or target proteins. However, on the downside, by design this library excludes compounds not causing the selected growth-related phenotypes but nonetheless may impair novel and/or important functions.

Closely related to selecting the chemical library is the question about the concentration to be used in the screening. There is no general answer to this question, but a few things need to be considered. Most commonly, chemical libraries are provided as 10 mM stock solutions solved in DMSO. For most bioassays performed with *Arabidopsis* seedlings, final DMSO concentrations of 1–5% can be tolerated, which puts an upper limit to the screening concentration at 100–500 μM. However, at high concentrations many chemicals may be toxic or cause stress responses thereby increasing the risk of generating many false positive (or false negative) hits. Although performing the screening at various concentrations would be the ideal solution, this approach requires additional effort, time, and costs. Typically, this is affordable only in commercial research programs employing robotic systems for handling microplates, dispensing fluids and determining activity in very robust and reliable bioassays, with the advantage of directly generating the half maximum effective concentration (EC_50_) values from a chemical screen (Miller et al., 2012). In plant research, as in other systems, the initial screening is carried out at a fixed concentration, which largely depends on the type of bioassay and the chemical library. HTS in drug discovery usually use low concentrations in the micromolar or nanomolar range, since high concentrations generate more hits, which require more effort for validation and effective compounds that are active at low concentrations are more desirable. In addition, compounds with high activity represent useful lead structures that could be used for chemical optimization and synthesis of more effective drugs (Landro et al., 2000). With respect to the bioassay, it is worthwhile to consider that in target based screening approaches carried out *in vitro*, the compounds typically show higher potency because they have direct access to the target without restriction by membranes or other barriers. On the other hand, phenotypic screening *in vivo*, employing cells or whole organism often require higher concentrations because the chemicals have to cross membranes or other barriers and might require transport to different organs or cellular compartments for activity. Another issue not to be neglected is the stability of compounds and metabolic conversion to active (or inactive) products, which is more likely to occur in complex systems such as cell-based assays. Chemical screenings performed in plant systems have employed a wide range of concentrations (2-200 micromolar) with the majority of screenings restricting the range to 20–50 micromolar (see Supplementary Table 1). One should not get too excited about hits that require high concentrations of the compound, such as 200 micromolar or more. As already stated by Paracelsus (1493–1541), the founder of modern toxicology and medicinal chemistry, “the dose makes the poison” (Borzelleca, 2000) and hence using relatively high concentrations bears the risk of obtaining false positive or negative hits (depending on the type of assay), as a result of stress responses to inappropriate cytotoxic concentrations. Thus, especially libraries that are enriched in bioactive compounds (e.g., Bioactive, Natural Product, and DOS/BIOS collections) can be used even at lower concentrations (10-25 micromolar) to avoid numerous unspecific hits. Conversely, libraries of high chemical diversity (RO5 and fragment collections) can potentially be screened at higher concentrations (~100 micromolar). Compounds with weak activity identified from such screenings, can often be converted to more active derivatives by chemical modification, yielding valuable information about the structure–activity relationship (SAR).

An interesting approach for reducing time and effort that is needed for library screening is to use pools of compounds (Devlin et al., 1996). Individual chemicals are combined in such a way that each is contained twice in unique compound pools. Screening of these pools creates unique distribution patterns for each component of the pools, which allows identification of an active compound by its pattern without the need to re-analyze each member individually. Obviously, this strategy relies on the assumption that the majority of compounds is inactive in a given bioassay that is sufficiently robust and sensitive. However, there are also certain caveats associated with this approach: (1) The combination of compounds may eventually lead to lower applicable concentrations (considering an upper limit of solvent that can be applied), which may only allow the identification of potent compounds; (2) molecular interaction between compounds may affect their stability and their activity (Hann et al., 1999); (3) false positive or negative hits may originate from additive or opposite biological activity of compounds in the same well. Although compound pooling has been successfully applied for chemical screening in a plant system (Tsuchiya et al., 2010), it has to be carefully considered whether or not it offers a true advantage.

### Verification and validation of hits

After hits have been selected from the primary screening, the next essential step is to rigorously validate the compound's biological activity and establish whether or not they selectively impair only one particular phenotypic readout (Figure 2). The first step is to repeat the screening assay with the selected hits to eliminate false positives. False negatives can only be avoided by screening in replicates. Missing an active compound may be annoying, but may be irrelevant if a sufficient number of positive hits has been identified. Again, a robust bioassay and application of stringent selection criteria are key to identifying strong candidate compounds. It is long been known that the dosage of a chemical affects the quantity of a response (Hill, 1910). Therefore, determination of rough pharmacodynamics by using various concentrations should at least be considered to re-evaluate the selected primary hits that would convey information about dose dependency and increase confidence in hit selection.

In order to establish reliable dose-response relationships, it is necessary to have a quantitative readout. However, even if a non-quantitative phenotypic readout is used for screening, it may be quantifiable in subsequent, individual bioassays. For example, hypocotyl length of seedlings visually inspected in chemical HTS can be quantified for individual compounds (Gendron et al., 2008; Savaldi-Goldstein et al., 2008; De Rybel et al., 2009; Lin et al., 2010; He et al., 2011). Likewise, GUS activity in HTS by staining, can be quantified *in vitro* by enzymatic conversion of the substrate 4-methylumbelliferyl-β-D-glucuronide to fluorescent 4-methylumbelliferone (Armstrong et al., 2004; Serrano et al., 2007; Knoth et al., 2009). An important parameter for evaluating a drug or chemical is the half-maximum effective concentration (EC_50_), or for inhibitors, the half-maximum inhibitory concentration (IC_50_) (Holford and Sheiner, 1981). For accurate EC_50_/IC_50_ calculation, it is essential to include sufficient assay concentrations to accurately determine both the maximal and minimal effective concentration (Sebaugh, 2011). Once the EC_50_/IC_50_ value is established, subsequent experiments for characterization of a compound can be carried out at a defined EC_50_/IC_50_, avoiding adverse effects at unnecessarily high concentrations at which the compound may be toxic or impinge on unrelated biological readouts.

The second step in validation of primary hits should be an independent bioassay from the same signaling pathway to confirm the chemical's biological activity by an alternative readout, e.g., a different reporter or quantifying endogenous gene expression. Such secondary assays are also referred to as orthogonal assay (Malo et al., 2006) and depending on the screening design and library size, it could be directly integrated into the primary screening, which is then performed with two different readouts in parallel or one after the other (Gendron et al., 2008; Tsuchiya et al., 2010; Nishimura et al., 2012, 2014; Hu et al., 2014). The toxicity of chemicals is also an issue that should not be neglected. To exclude that induced cell death interferes with the biological readout, cell death should be monitored upon chemical treatment separately or, if the bioassay allows, as integral part during the recorded readout (Noutoshi et al., 2012). In reporter-based screenings, the potential interference of a chemical with the reporter activity also needs to be considered. For example, 2-3 percent of a chemical library typically interfere with luciferase activity and in addition, two percent of the same library usually exhibit fluorescence at a similar wavelength as 4-methylumbelliferone, which is the frequently used substrate for quantitative measurement of GUS activity (Inglese et al., 2007). Correspondingly, reporter-based screening results need to be verified by appropriate counter assays to eliminate false positives. Dual or single reporter lines harboring different reporters under the control of the same promoter represent excellent tools, but any other control is also appropriate, such as monitoring endogenous gene expression (Meesters et al., 2014).

Another important step during characterization of a bioactive agent is to evaluate the compound's selectivity. The ideal chemical tool affects only a single target, which is an essential component of the studied biological process; it does not interact with secondary sites, so-called off-targets and thus has no side effects. In pharmacology, such selectivity is highly desirable because it facilitates registration and marketing of a drug. Early stage identification of possible off-targets can reduce time and costs and an extensive characterization may prevent drugs from been withdrawn from the market (MacDonald et al., 2006; Hughes et al., 2011). Of course, basic research is not restricted by such regulation, but generally, target-selective small molecules are superior chemical tools.

To establish selectivity of a candidate compound, its impact on numerous independent biological readouts needs to be tested. Such counter assays can easily be performed with transgenic reporter lines that respond to different stimuli. But in fact, any assay that is independent of the screened phenotype would be suitable. However, it is also important to bear in mind, that some signaling pathways share similarities in their perception and signaling mechanisms or cross-talk with each other, as recently demonstrated for the plant hormones auxin, gibberellin, jasmonate and salicylic acid (SA) (Katsir et al., 2008; Pieterse et al., 2009; Santner and Estelle, 2009; Vlot et al., 2009; Lumba et al., 2010). Thus, also the selection of bioassays to be used for counter screening needs careful consideration to avoid pitfalls. Although selective chemicals are preferable, even non-selective compounds may be of value. For example, three non-selective and mechanistically distinct inhibitors of germination (cycloheximide, methotrexate, and 2,4-dinitrophenol) were applied in a comparative microarray study to uncover the common genes that are exclusively involved in germination (Bassel et al., 2008).

Consulting chemical databases (e.g., ChEMBL, PubChem) for retrieving information about primary hits may facilitate the validation process considerably. Much of this information originates from other screening campaigns, predominantly from animal systems and drug discovery programs, but still, this information may point to potential targets and indicate whether a compound is selective or affects various processes. Along the same lines, it should be considered to use the same chemical library for multiple screenings with different biological readouts, therefore enabling easy validation by comparing the results of independent screening campaigns. Such parallel independent screenings provide the instant possibility to filter out the compounds with unique or common activity profiles, which eventually may save efforts and costs for subsequent compound validation. Another advantage of chemical databases is the possibility to search for structural derivatives and their bioactivities. Such derivatives of a candidate compound are important for studying the SAR, which may lead to a panel of compounds with different specific activities. The knowledge of the SAR may also be crucial for subsequent target identification strategies, because it may identify the site(s) of a molecule that tolerates modifications without loss of activity and inactive analogs may serve as useful control in biochemical target identification strategies (Meesters et al., 2014).

Finally, it should not be ignored that validation of a bioactive compound identified from a chemical library should always include verification of its chemical identity and purity. Eventually, it may be necessary to re-synthesize the compound if no alternative source or provider can be identified.

### Target identification

Once a small molecule has been selected from the chemical screening, the molecular target needs to be identified in order to fully understand the compound's effect on the biological system. However, target identification is usually the limiting step of a chemical genetic project. This is mainly due to three limitations, but not restricted to these: (1) Weak and reversible interaction between the small ligand and its protein target (i.e., low binding affinity); (2) low abundance of the target (or multiple targets); (3) adverse, intrinsic properties of the small molecule, e.g., lack of suitable functional groups preventing appropriate chemical modification (i.e., introduction of a tag) or impaired activity after such modification (Burdine and Kodadek, 2004; Zheng et al., 2004; Walsh and Chang, 2006; Terstappen et al., 2007). In chemical biological research, different technological approaches have been successfully applied to identify small molecule targets. In this section, we will briefly describe few examples and provide an overview of possible target identification strategies, which include genetic screening, biochemical affinity purification, proteomic methods, and DNA-based approaches. For a more detailed discussion, we refer to review articles focusing on this topic (Tashiro and Imoto, 2012; Schenone et al., 2013; Ziegler et al., 2013; Dejonghe and Russinova, 2014).

A generally applicable target identification methodology is forward genetic screening; in fact, the integration of small molecules into genetic strategies specifically defines “chemical genetics.” Essentially, the genetic screening part aims at identification of mutants that escape from the chemically induced phenotype. Such mutants that are either insensitive or hypersensitive to previously identified compounds are used for genetic mapping, because the corresponding locus (or a closely associated component) is likely a direct target. In the past, physical mapping of a mutation was time-consuming and labor-intensive. However, with the advent of new sequencing technologies such as next-generation sequencing (NGS) the rapid and cost-effective identification of mutations by whole-genome sequencing has been made possible (Schneeberger et al., 2009). With millions of short reads that are generated from F2 mapping populations using NGS platforms (e.g., Ilumina Genome Analyzer) the distribution of the single nucleotide polymorphism (SNPs) between the reference (i.e., the corresponding wild-type) and mutant genomes are analyzed. Using this methodology the number of candidate genes causing the mutant phenotype can be narrowed down in a rather short time period (Schneeberger et al., 2009; Austin et al., 2011).

**Figure 5:**
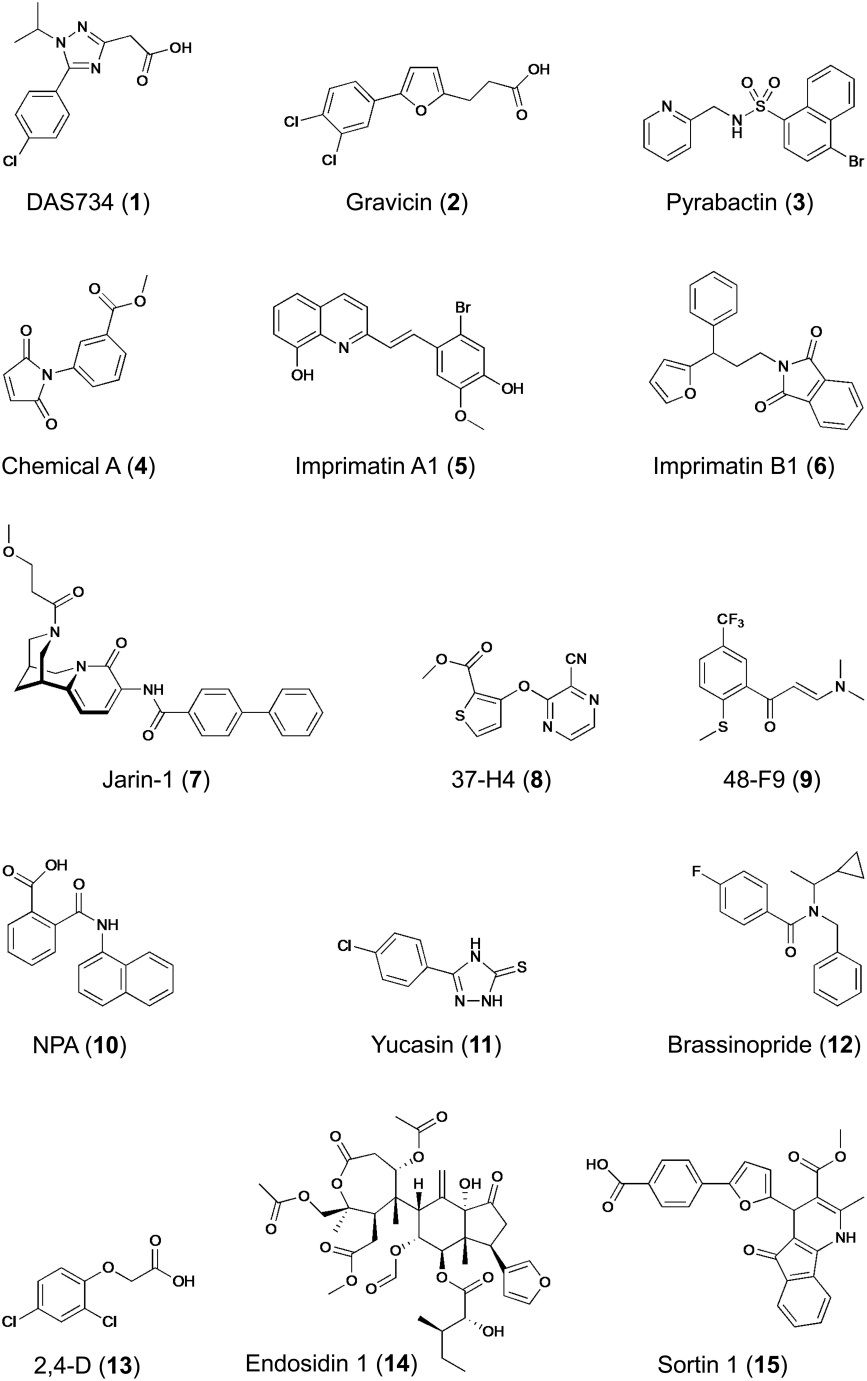
**Structures of bioactive compounds identified in different chemical screening campaigns**. Examples refer to compounds mentioned in this paper.

Forward genetic screenings have been successfully used in plant chemical biology. For example, glutamine phosphoribosylamidotransferase (AtGRAT2) has been identified as target of the novel herbicide DAS734, a phenyltriazole acetic acid derivative (**1**) (Figure 5), thereby establishing its utility as a new and specific inhibitor of plant purin biosynthesis (Walsh et al., 2007). Similarly, P-glycoprotein19 (PGP19), a member of the superfamily of ATP-binding cassette (ABC) transporters was shown to bind gravicin (**2**), which was identified in a chemical screen for inhibitors of gravitropism and functions by selectively impairing auxin transport activity of PGPs but not that of other auxin transporters such as PIN proteins (Rojas-Pierce et al., 2007). Most remarkable, however, is the identification of the abscisic acid (ABA) receptor by combined chemical and genetic approaches (Park et al., 2009). From a chemical screening for seed germination inhibitors, the small molecule pyrabactin (**3**) (Figure 5) was identified, which induced phenotypes resembling ABA treatment (e.g., activation of ABA-responsive genes), thus acting as an ABA agonist (Zhao et al., 2007; Park et al., 2009). However, mutants isolated by genetic screening for pyrabactin insensitivity were not resistant to ABA. The identified causal gene *PYRABACTIN RESISTANCE 1* (*PYR1*), encodes a member of the superfamily of proteins containing the so-called START domain, which is important for binding and transfer of lipids; other members of this superfamily, referred to as PYR1-LIKE (PYL) or REGULATORY COMPONENTS OF ABA RECEPTORS (RCAR) were identified as interactors of *ABA-INSENSITIVE 1/2* (*ABI1/2*) encoding type 2C protein phosphatases (PP2Cs), which function as negative regulators of ABA signaling (Ma et al., 2009; Park et al., 2009). Importantly, PYR1/PYL and PP2Cs act as a family of ABA co-receptors forming a ternary complex with ABA, which results in inhibition of PP2C activity and initiation (de-repression) of downstream responses, including activation of ABA responsive genes (Ma et al., 2009; Melcher et al., 2009; Miyazono et al., 2009; Park et al., 2009; Cutler et al., 2010). The identification of the long-sought ABA receptor is an outstanding example, among others (Hicks and Raikhel, 2014), demonstrating the power of chemical genetics to circumvent gene redundancy as pyrabactin selectively activates only one out of 14 PYR1/PYL proteins, a property that is distinctly different from ABA (Cutler et al., 2010).

Biochemical *in vitro* purification methods using labeled small molecules are the traditional and direct approaches for target identification (Schenone et al., 2013; Ziegler et al., 2013). However, this methodology suffers from severe limitations. While target identification of chemically reactive small molecules *via* affinity purification and proteomics has become routine (Wang et al., 2008; Kaschani et al., 2009), target identification of relatively inert small molecules (i.e., non-covalent binding ligands) remains challenging. Introduction of tags for photoaffinity cross-linking, immobilization on a solid support or radio-labeling requires prior knowledge of SAR to retain biological activity and, of course, the presence of suitable functional groups. In addition, these modifications (as well as the subsequent purification steps) may be labor-intensive and time-consuming. Further difficulties are encountered when targets are present in low abundance, as is often the case for membrane-localized receptors, or the ligand shows only low binding affinity (Burdine and Kodadek, 2004; Terstappen et al., 2007). To circumvent these and related problems, alternative profiling and target identification strategies have been invented, many of which are sophisticated and/or technically challenging (Lomenick et al., 2009, 2011; Rix and Superti-Furga, 2009; Schenone et al., 2013).

A new profiling technique to identify the protein target (or targets) at the proteome scale without the necessity to modify the corresponding small molecule is the drug affinity responsive target stability (DARTS) method (Lomenick et al., 2009; Aghajan et al., 2010). The DARTS method is based on the thermodynamic stabilization of the protein target upon binding of the small molecule, which renders the protein less prone to degradation by proteases in comparison to non-bound proteins. The advantage of this approach is that it can be performed in crude extracts without prior protein purification and that target identification is label free. Although the DARTS method is restricted to abundant targets, signal loss is limited as no washing steps are required. Alternatively, target deconvolution can be achieved by RNA profiling technologies (DNA-microarray analysis) or other genomic approaches (Terstappen et al., 2007; Schenone et al., 2013; Ziegler et al., 2013). Analyzing transcriptional changes in response to a chemical, using DNA microarrays or RNA-seq, allows identification of a molecule's molecular signature, which can be compared with preexistent transcriptional profiles of collections of mutants or caused by other compounds. However, this approach has several limitations: (1) The target needs to mediate a transcriptional output, (2) it requires the prior existence of such molecular signatures (e.g., for drug discovery such profiles may be available from databases), and (3) it does not relieve from extensive characterization of target candidates by ligand binding assays (Stockwell, 2000; Walsh and Chang, 2006).

Another set of methods have in common that identification of a small molecule target is combined with cloning of its cDNA (Terstappen et al., 2007; Schenone et al., 2013). Such expression cloning technologies, including the yeast three-hybrid (Y3H) system, phage display and mRNA display, artificially increase the abundance of the target by expressing it as recombinant fusion protein, which may have properties that are different from the native original, in particular, when post-translational modifications are involved. Among these techniques, the Y3H system is particularly appealing because it not only offers direct access to the genes encoding target proteins, but it also relies on small molecule–protein interactions in living cells rather than *in vitro* and it permits scanning of whole proteomes for targets (Kley, 2004; Terstappen et al., 2007; Cottier et al., 2011). Importantly, this approach is not restricted to model organisms. The Y3H technology, originally developed by Licitra and Liu (1996), is an extension of the commonly used yeast two-hybrid system by introducing a third hybrid component, the small molecule of interest linked to another ligand, usually methotrexate or dexamethasone (Cottier et al., 2011). The functional output of the small molecule (as part of the hybrid ligand) binding to its cDNA encoded protein target is growth of the corresponding transformed yeast cell to a colony, which will serve to directly identify the binding protein by cDNA sequencing. Of course, this promising technology also suffers from limitations: (1) The functional readout of the system is gene activation and therefore only soluble proteins that are translocated to the yeast nucleus are detectable (e.g., excluding membrane-localized receptors), (2) identification of multimeric protein complexes is not possible because only single cDNAs are expressed in individual yeast cells, and (3) uptake of hybrid ligands (as they are relatively large molecules) may be impaired or excluded as yeast has efficient drug extrusion systems.

Another novel and sensitive technology to determine ligand–target interaction is the analysis of a protein microarray using the surface plasmon resonance (SPR) imaging (SPRi). The SPR technology records changes in light refraction on sensor chip surfaces that occur upon interaction between two (or more) binding partners, one of which is covalently linked to the sensor chip surface. SPRi is the current leading technology for label-free detection of protein interactions and a powerful tool for affinity-based biosensors in high throughput screens (Hall et al., 2007; Ray et al., 2010). Instead of linking only one particular protein to the sensor chip, proteins originating from cDNA libraries ideally representing the whole proteome (Yamada et al., 2003; Gong et al., 2004) could be spotted onto the protein microarray to detect the interaction partner of the compound of interest. Additionally, the recent development of nanohole arrays increases spatial resolution, facilitating the development of protein arrays (De Leebeeck et al., 2007). Advantages of SPRi-protein array analysis are that even natural low abundant proteins are detectable and that it enables kinetic characterization of the protein–ligand interaction (Rich et al., 2002). In addition, it can be used to identify not only the main target of a small molecule, but also off-targets with weaker interaction (Lomenick et al., 2011). However, identification of small molecule targets using proteome arrays is an unexplored field in plant sciences.

## Chemical screenings in plant biology

In the past decade, plant chemical biology has seen substantial progress with more than thirty performed chemical screenings analyzing various biological processes (see Supplementary Table 1). After having discussed the conditions and recommendations for performing such chemical screenings, we will now present a few striking examples and highlight their distinct characteristics.

### Target-based approaches

In plant chemical biology, there are only two examples of target-based chemical screenings. In the first example Yoshitani and colleagues were interested in finding specific ligands of an *Arabidopsis* protein in order to unravel its unknown function (Yoshitani et al., 2005). This study combined *in silico* screening based on the protein's three-dimensional structure with subsequent evaluation of candidate compounds using immobilized recombinant protein in a SPR assay. From a chemical database, 103,773 compounds were taken for *in silico* screening. Two rounds of molecular docking to a predicted ligand-binding site identified 10,000 and 300 top scoring compounds, respectively. Out of the best scores, 69 compounds were subsequently analyzed for their binding properties at the molecular level. Four compounds showed weak interactions with the recombinant protein and all shared common structural features, suggesting that these determine their affinity to the target protein. However, the protein function remains elusive, but the compound's common structure can serve as a lead for the development of specific inhibitors or may provide important clues toward elucidation of the protein function. Essentially, this is a proof of concept that computational screening in combination with SPR-based experimental evaluation can discover candidate ligands or substrates. Clearly, this approach depends on the correct prediction of a potential binding site; it cannot be applied for proteins undergoing structural changes upon ligand binding. There are several studies with non-plant proteins that provide evidence for virtual screening as effective tool for identifying protein function (Kalyanaraman et al., 2005; Hermann et al., 2007; Song et al., 2007; Mallipeddi et al., 2012). The advantage of combining *in silico* with experimental screening is that the virtual pre-selection of compounds can dramatically reduce time, effort and expenses associated with experimental screening.

The second example of target-based screening relates to the biosynthesis of plant cell wall polysaccharides (Zabotina et al., 2008). The synthesis of highly complex polysaccharides constituting the plant cell wall is thought to involve at least 1000 genes and biochemical changes caused by mutations create only weak phenotypes difficult to discern (Somerville et al., 2004). Therefore, a chemical biology approach seemed appropriate. Most enzymes involved in the synthesis of extracellular polysaccharides are located in the Golgi apparatus and therefore, Zabotina and colleagues monitored the conversion of radiolabeled UDP-glucose in isolated pea stem microsomal fractions. This quantitative *in vitro* screening led to identification of ten compounds (out of 4800 screened) that inhibited the incorporation of glucose into cell wall carbohydrates. Remarkably, chemical A (**4**) (Figure 5) not only inhibited Golgi-localized glucosyltransferase activity, but also modified cell wall composition *in planta* and activated plasmamembrane-bound callose synthase without affecting the endomembrane morphology (Zabotina et al., 2008). Chemical A represents a novel drug with great potential for the study of the mechanisms of Golgi and plasmamembrane-bound glucosyltransferases and a useful tool for identification of additional enzymes involved in polysaccharide biosynthesis. Despite the presence of additional enzymes in the assay that could be molecular targets, one can classify this screening as target-based due to the fact that a specific substrate was used, which drove the assay toward identification of effectors of proteins capable of using this particular nucleotide sugar as substrate.

### Phenotypic approaches

As mentioned earlier, the majority of the chemical screenings performed in plant systems are forward or phenotypic screenings using a qualitative readout. Despite the obvious advantages of quantitative screening assays, only few examples exist for this superior strategy (Supplementary Table 1). Noutoshi and colleagues performed such a quantitative chemical screening with cultured *Arabidopsis* cells aiming at the identification of compounds that enhance disease resistance by specifically potentiating pathogen-activated cell death (Noutoshi et al., 2012). This study was inspired by the fact that exogenous application of SA (and related compounds that even have practical applications) confers disease resistance to plants (Kessmann et al., 1994; Schreiber and Desveaux, 2008; Bektas and Eulgem, 2014). Out of 10,000 diverse chemicals, five compounds were identified that increased cell death upon challenge with pathogenic *Pseudomonas* bacteria but that were not toxic by themselves (up to concentrations of 100 μM). Importantly, *Arabidopsis* cell death was quantified by Evans blue staining in three replicates and selected candidates were subjected to a dose–response analysis, which provided a high confidence of hit selection. The identified compounds represented two distinct molecular structural backbones, which were designated imprimatins A (**5**) and B (**6**) (Figure 5) for immune-priming chemicals. Remarkably, the immune-priming effect was also effective in *Arabidopsis* seedlings as treatment with imprimatins enhanced resistance to bacterial infection. Further characterization of the compounds revealed that pretreatment with imprimatins increased the accumulation of endogenous SA, whereas its metabolite, SA-*O*-β-D-glucoside, was reduced. This is the result of the selective inhibition of two SA glucosyltransferases (SAGTs) as demonstrated by *in vitro* enzyme assays. In addition, loss of function mutants of these two SAGTs phenocopied the effect of imprimatins, indicating that SAGTs are involved in immune priming by modulating the pool of free SA. Considering potential application, the results of this study demonstrate that manipulation of the active free SA pool *via* SA-inactivating enzymes could be a useful strategy for fortifying plant disease resistance and may lead to novel and useful crop protectants. However, whether the protection conferred by these compounds is as durable as that of other plant activators remains to be established (Noutoshi et al., 2012; Bektas and Eulgem, 2014).

Another example of employing a quantitative chemical screening strategy has recently led to the identification of a selective inhibitor of jasmonate signaling (Meesters et al., 2014). *Arabidopsis* seedlings harboring a jasmonate-inducible luciferase-based reporter system allowed facile screening for inhibitors of jasmonate-induced gene expression by *in vivo* monitoring of luciferase activity. Although the quantified *in vivo* luciferase luminescence showed considerable variation resulting from differences in seedling size and orientation in microplate wells, the method impresses by its simplicity and yielded several candidate inhibitors from a small library of approximately 1700 compounds of natural and semi-synthetic origin. Rigorous validation of the identified candidates by orthogonal and counter assays uncovered jarin-1 (**7**) (Figure 5) as selective inhibitor of different jasmonate-dependent phenotypes (Meesters et al., 2014). The cognate target of jarin-1 was identified by systematic scanning of all known components participating in jasmonate biosynthesis and signaling, eventually establishing that jarin-1 binds to and inhibits the activity of jasmonoyl-L-isoleucine synthetase, encoded by *JASMONATE RESISTANT 1* (*JAR1*), which catalyzes the conjugation of jasmonic acid (JA) with L-isoleucine to the bioactive form of the hormone, (+)-7-*iso*-JA-L-Ile. Notably, JAR1 is the only member of the large family of adenylate-forming enzymes, conjugating several plant hormones (e.g., auxin, SA, JA) with amino acids, that is impaired by jarin-1 (Meesters et al., 2014). As this inhibition is effective not only in *Arabidopsis* but also in other plants, jarin-1 could prove a useful chemical tool for jasmonate research. Collectively, this study provides an outstanding example of a complete chemical genetic procedure, including hit selection by quantitative screening, verification and validation of primary hits by orthogonal and counter assays, SAR studies, and finally identification and characterization of the selective compound's molecular target.

In contrast to quantitative screenings, qualitative screenings may lead to biased hit selection, as phenotype evaluation is then prone to subjective decisions. To increase the confidence in hit selection, one possibility is to use multiple readouts. Essentially, this approach combines primary screening with first hit validation in one step, thereby helping to eliminate compounds that have pleiotropic effects. Plant hormones participate in multiple biologic processes and to circumvent their pleiotropic responses, several chemical screenings focusing on responses caused by plant hormones (e.g., auxin, strigolactone, or ethylene) have utilized such multiple readouts (Tsuchiya et al., 2010; Nishimura et al., 2012, 2014; Hu et al., 2014). For example, in search for new auxin transport inhibitors two parallel screenings were applied to the same chemical library of 10,000 compounds: (1) monitoring the gravitropic curvature of maize coleoptiles, and (2) determination of indole-3-acetic acid (IAA) transport in coleoptile segments (Nishimura et al., 2012). Further characterization of eight candidate compounds originating from both screens eventually led to the identification of two new inhibitors of IAA transport [e.g., 37-H4 (**8**) and 48-F9 (**9**) (Figure 5)] that are structurally different to the known auxin transport inhibitor 1-*N*-naphthylphthalamic acid (NPA, **10**), and therefore represent novel tools for dissecting the mechanism of auxin transport in plants. In a follow-up analysis, the same screening approach was used to identify inhibitors of IAA biosynthesis (Nishimura et al., 2014). As three selected compounds shared structural features with methimazole, an artificial substrate for flavin-containing mono-oxygenase (FMO), it was postulated that they may target YUCCA (YUC), a plant FMO (or FMO-like) protein that participates in IAA biosynthesis by catalyzing the hydroxylation of the amino group of tryptamine (Dai et al., 2013). The most potent inhibitor, yucasin (**11**) (Figure 5), was confirmed to impair the activity of recombinant *Arabidopsis* YUC1 protein *in vitro* and was further shown to suppress the high-auxin phenotype of plants overexpressing *YUC1*. However, yucasin did not affect IAA-dependent gene expression or auxin signaling after exogenous application of IAA (Nishimura et al., 2014). Thus, yucasin was shown to be a potent inhibitor of YUC enzymes *in vitro* and *in planta* and a useful tool in the quest for missing components of auxin biosynthesis and signaling.

Similarly, sequential screening for two different phenotypes was also successfully applied to find new inhibitors of brassinosteroid (BR) action (Gendron et al., 2008). Several chemical inhibitors of BR synthesis had previously been identified (Izumi et al., 1985; Asami et al., 2000, 2003, 2004; Sekimata et al., 2001, 2002) and their application in suppressor screens uncovered novel components of BR signaling (Wang et al., 2002; Yin et al., 2002, 2005; He et al., 2005). In search for novel inhibitors of BR signaling/synthesis, the retarded hypocotyl-length of dark-grown *Arabidopsis* seedlings served as first selection criterion, as inhibition of BR action causes dwarfism. Seedlings of a transgenic *Arabidopsis* line harboring the BR-repressed *CPD::GUS* reporter showing short hypocotyls upon treatment with chemicals were subsequently monitored for *GUS* expression as second indicator of reduced endogenous BR levels (Gendron et al., 2008). By this approach, chemicals impairing growth either directly or indirectly (e.g., by affecting other hormonal pathways) were easily eliminated. As result of this stringent selection scheme, only one unique inhibitor of BR biosynthesis, brassinopride (**12**) (Figure 5), was identified from a library of 10,000 diverse chemicals. The structure of brassinopride is quite different from other known BR inhibitors and physiological experiments further showed that it not only affected BR biosynthesis but also activated the ethylene signaling pathway (Gendron et al., 2008). Although this study did not uncover a direct target of brassinopride, it provided new insight into BR and ethylene cross-talk in seedling development. Another chemical screen monitoring also hypocotyl length aimed at identification of growth promoting compounds (Savaldi-Goldstein et al., 2008). Taking advantage of a BR-deficient *Arabidopsis* dwarf mutant, thereby facilitating the phenotypic analysis, 100 out of 10,000 compounds screened were found to promote hypocotyl length (Savaldi-Goldstein et al., 2008). Rather than performing extensive verification and validation of all compounds, the authors chose to search for common structural features and identified several compounds that share high similarity to the synthetic auxin, 2,4-dichlorophenoxyacetic acid (2,4-D, **13**) (Figure 5). Remarkably, auxin had not been previously reported to directly affect hypocotyl length of light-grown seedlings. The effect of these synthetic proauxins on hypocotyl length was explained by efficient absorption and diffusion into this organ, where they undergo cleavage to functional auxins. Indeed, the compounds satisfied the Lipinski's RO5, they have a high probability of facile diffusion across cell membranes, and when incubated with seedlings, they liberated auxin- and 2,4-D-like molecules. Thus, the chemical biological approach has led to the discovery of novel proauxin analogs with selective activity in specific plant tissues (Savaldi-Goldstein et al., 2008). This example illustrates the need to consider various aspects associated with a compound's bioactivity, including uptake (facilitated for RO5 compounds), translocation or chemical modification (metabolism, detoxification) as it may occur within the cells/organism.

The commonly used plant systems for chemical screenings are seedlings or cultured cells, but particular biological processes may require other systems that suit better the needs for studying the process of interest. For example, proteins are delivered to and recycled from the plasmamembrane *via* endosomes, but the process and pathways of vesicle and cargo sorting is poorly understood and chemical modulators of vesicle trafficking are therefore desirable. The process of unidirectional (or polar) cellular growth involves intense vesicle trafficking and in plants this is obvious especially in root hairs and pollen tubes (Cole and Fowler, 2006). To identify chemicals affecting essential steps in plasmamembrane–endosome trafficking, Robert and colleagues designed an automated image-based screening with tobacco pollen by microscopic monitoring germination and tube morphology, which are both dependent on vesicle transport (Robert et al., 2008). Although only 2016 chemicals were screened, several bioactive compounds were identified, including cantharidin, a protein phosphatase inhibitor previously shown to affect the localization of auxin transporters (thus providing a proof of concept for the screen), and endosidin1 (ES1, **14**) (Figure 5), which interfered selectively with endocytosis not only in pollen but also *Arabidopsis* seedlings. In fact, ES1 treatment blocked the endocytosis of several auxin transporters (PIN2, AUX1), which are known to recycle in *Arabidopsis* roots, as well as the brassinosteroid receptor BRI1, leading to a brassinosteroid-insensitive phenotype, thereby demonstrating that all three plasmamembrane-resident proteins share overlapping endocytic pathways (Robert et al., 2008). Two additional findings are important in this context. First, the automated image-based phenotyping is suitable for high-throughput screening, as demonstrated by a subsequent report extending the approach to high-content intracellular image analysis using more than 46,000 compounds (Drakakaki et al., 2011). Second, an independent chemical screening for effectors of the circadian clock in *Arabidopsis* seedlings also identified ES1 (**14**), and subsequent work showed that ES1 treatment stabilized the actin cytoskeleton *in vivo*, which caused changes in vesicle trafficking (Tóth et al., 2012). The identification of the actin-stabilizing effect was facilitated by comparing the effect of the compound on plant development to mutant phenotypes and to other drug treatments. Remarkably, ES1 also affected microfilaments in mammalian cells, indicating that its target is highly conserved. Thus, ES1 affects rhythms (i.e. period length of the clock) and endosome trafficking by altering the actin network. As it differs from previously described inhibitors, it may be a useful tool for studying actin-related processes.

For studying fundamental processes in plants, it may be useful to initiate work in a different simplified biological system. Trafficking of endomembranes is evolutionarily conserved and a cell autonomous process and therefore the unicellular eukaryote yeast, *Saccharomyces cerevisiae*, was employed as a substitute for a plant-based system to identify chemicals affecting the endomembrane system (Zouhar et al., 2010). A further rational for this approach lies in the fact that vacuolar biogenesis is an essential process in plants and mutants lacking proper vacuole development are embryo lethal (Rojo et al., 2001). Therefore, using yeast grown in 96-well microplates, a library comprising 4800 diverse chemicals was screened for compounds that caused secretion of carboxypeptidase Y (CPY), which is normally targeted to the vacuole (Zouhar et al., 2010). One of several identified protein-sorting inhibitors, named sortin1 (**15**) (Figure 5), was also active in *Arabidopsis* seedlings, causing reversible root growth inhibition and secretion of the plant CPY. Remarkably, sortin1-hypersensitive *Arabidopsis* mutants exhibited severe vacuolar morphology phenotypes and also showed defects in flavonoid accumulation (Rosado et al., 2011). Although the cognate target of sortin1 is not yet known and the mechanism of transport and vacuolar accumulation of flavonoids likewise remains unclear, sortin1-hypersensitive mutants and sortin1, as well as structural derivatives, will be useful tools to shed more light on vacuolar biogenesis and flavonoid transport in *Arabidopsis*. Again, these results clearly demonstrate the power of the chemical screening approach for identifying novel plant-active compounds affecting the endomembrane system in plants, which has proven difficult to dissect by conventional genetics.

### Exploring new experimental systems

The central feature of all chemical screening projects is a miniaturization bioassay that is suitable for automated HTS. Most chemical screenings in plant systems have so far been conducted with *Arabidopsis* seedlings grown in microplates. Other systems such as cultured cells, pollen tubes germinated *in vitro*, or yeast cells (as heterologous substitute) have also been applied successfully, but not every pertinent biological question can be adapted to the microplate format. For example, automated systems for the analysis of root architecture have been reported (Armengaud et al., 2009; Ingram et al., 2012; Wells et al., 2012). These systems are not miniaturized and therefore chemical treatment would be difficult and expensive to perform. However, with a special effort Forde and colleagues developed a customized microplate system for high-content automatic image analysis of root architecture in *Arabidopsis* seedlings, which can be combined with chemical treatment (Forde et al., 2013). This provides a good example that even uncharted biological territory can be made accessible to interrogation by chemical biology. But there are still numerous plant processes that are recalcitrant to exploitation by the potential of chemical biology such as flowering, which is commonly associated with mature and large-size plants that cannot be hosted in microplates. As a substitute, duckweeds (Lemneae and Wolffieae sp.), which include the smallest flowering plants known, can easily be grown in liquid medium in microplates and were previously suggested to serve as model systems for studying flowering even before the emergence of *Arabidopsis* as model plant (Maheshwari and Chauhan, 1963; Kandeler, 1984). Indeed, it was shown that flowering of this aquatic plant can be controlled by application of chemicals such as SA, nitric oxide (NO) or cytokinin (Maheshwari and Venkataraman, 1966; Venkatar et al., 1970; Khurana and Maheshwari, 1983; Khurana et al., 2011). Despite apparent differences in NO-mediated induction of flowering in the monocotyledonous plant *Lemna aequinoctialis* and the dicot *Arabidopsis thaliana* (Khurana et al., 2011), the small aquatic duckweeds bear great potential for serving as powerful model systems for diverse chemical screening projects ranging from microscopic to macroscopic phenotypes such as endomembrane trafficking and flowering control, respectively.

## Conclusions and perspectives

Research in plant chemical biology has gained enormous momentum during the past 10 years with more than 30 diverse chemical screening campaigns being published that resulted in the identification of a large number of novel bioactive small molecules representing useful chemical tools for further dissecting biological processes (Supplementary Table 1). So far, there is a certain bias for analyzing synthesis and signaling pathways related to phytohormones, which may be related to the fact that these are bioactive small molecules mediating drastic phenotypic alterations (Fonseca et al., 2014; Rigal et al., 2014). Conversely, this also indicates that there is still enormous scope for extending chemical screening projects into yet unexplored areas of biology. As noted previously, one such area is cell biology with the need to score for intracellular phenotypes such as membrane trafficking, which requires establishment of automated screening systems for image and video analysis (Hicks and Raikhel, 2009, 2014). Likewise, application of biosensors, capable of monitoring intracellular concentrations of small molecules, and selective dyes for staining subcellular structures should be part of this development (Mur et al., 2011; Swanson et al., 2011; Okumoto et al., 2012). Given the availability of large collections of fluorescent-tagged intracellular marker proteins as well as the abundance of miscellaneous reporter lines and mutants, *Arabidopsis* will remain the prevailing experimental system for plant chemical biology. Thus, a steady and extensive application of chemical genetic approaches can therefore be expected. However, a continuous challenge is to develop screening methods that are rapid, simple, and robust (Zhang, 1999; Halder and Kombrink, 2015). In addition, the full potential of quantitative data acquisition thereby allowing rigorous application of statistical tools for hit selection and validation has not yet been realized in the plant sciences, whereas this approach is routine in drug discovery programs (Malo et al., 2006, 2010; Swinney and Anthony, 2011). Finally, target identification remains the biggest challenge in all chemical biology projects and yet this step is indispensible for understanding a chemical's mode of action. Correspondingly, it is not sufficient to simply find new compounds with interesting bioactivities; rather we have to push harder to gain insight into the biological systems under investigation by application of chemical tools.

### Conflict of interest statement

The authors declare that the research was conducted in the absence of any commercial or financial relationships that could be construed as a potential conflict of interest.
