# Supplementary material for: Considerations for designing chemical screening strategies in plant biology
Source: Front Plant Sci. 2015 Apr 8;6:131. doi: 10.3389/fpls.2015.00131 (PMC4389374; doi:10.3389/fpls.2015.00131)
Supplement: Supplementary file 1 [file Table1.PDF]

Supplementary Table 1. Chemical screenings in plant biology. The table lists reported chemical screenings in plant biology. N.d.: not defined, References marked with an asterisk report quantitative screenings

Targeted process	System	Plate size	Library size	Library source	Final conc.	Readout	Reference*
Chemical library construction	Arabidopsis seedlings / zebrafish	96	≤1412	Synthesis of biaryl-containing compounds	10 µM	Microscopic observation of development	(Spring et al., 2002)
Unknown protein function	Recombinant protein of Arabidopsis	96	103,773	SPECS compound database	50 µM	Protein binding (SPR)	(Yoshitani et al., 2005)*
Auxin signaling	Arabidopsis seedlings	24	-	Culture extract from *Streptomyces diastatochromogenes* B59	0.1, 1, 5, 10 µM	BA3 GUS-reporter system	(Hayashi et al., 2001)
	Arabidopsis seedlings	96	10,000	ChemBridge DIVERSet	20 µM	BA3 GUS-reporter system	(Armstrong et al., 2004)
	Maize coleoptiles	96	10,000	Maybridge HitFinder	100/200 µM	Coleoptile gravitropism / IAA measurement	(Nishimura et al., 2012;Nishimura et al., 2014)*
Brassinosteroid signaling	*Lepidium sativum* seedlings	1	10	Synthesized triazoles	1 µM	Hypocotyl length	(Min et al., 1999)
	Arabidopsis seedlings	96	10,000	ChemBridge DIVERSet	20-40 µM	Hypocotyl length and CPDp::GUS reporter gene expression	(Gendron et al., 2008)
	Arabidopsis seedlings	96	10,000	ChemBridge DIVERSet	50 µM	Hypocotyl length, petiole length and bending, leaf shape and color	(De Rybel et al., 2009)
Ethylene signaling	Arabidopsis (*eto1-4*) seedlings	96	10,000	ChemBridge DIVERSet	50 µM	Hypocotyl length	(Lin et al., 2010)
	Arabidopsis (*eto1-2*, *ctr1-1*) seedlings	96	2,000	Microsource Spectrum	50-100 µM	Hypocotyl and root length	(He et al., 2011)
	Arabidopsis seedlings	96	12,000	ChemBridge DIVERSet	50 µM	Ethylene triple response and EBSp::GUS reporter gene expression	(Hu et al., 2014)
ABA/GA signaling	Arabidopsis ML1-FUS3 (*wg3-11*) seedlings	24	10,000	ChemBridge DIVERSet	12.5 µM Mix of 8 chemicals	Inhibition of cotyledon expansion and greening after germination	(Tsuchiya et al., 2010)
	Arabidopsis seedlings	96	9,600	ChemBridge DIVERSet	20-40	RAB18p::GFP reporter gene expression	(Kim et al., 2011)
Jasmonate signaling	Arabidopsis seedlings	96	1,728	Analyticon Discovery	25 µM	LOX2p::LUC reporter gene expression	(Meesters et al., 2014)*
Light & hormone response	Arabidopsis *det2-1* seedlings	96	10,000	ChemBridge DIVERSet	20-40 µM	Hypocotyl elongation	(Savaldi-Goldstein et al., 2008)
Circadian clock	Arabidopsis seedlings	96	720	Microsource NatProd	50 µM	GIp::LUC reporter gene expression	(Toth et al., 2012)*

Targeted process	System	Plate size	Library size	Library source	Final conc.	Readout	Reference*
Plant immunity	Arabidopsis seedlings	96	120	SYNGENTA bioactive compounds	10 ppm	ATL2p::GUS reporter gene expression	(Serrano et al., 2007)
	Arabidopsis seedlings	96	<200	LATCA	25 µM	Leaf bleaching	(Schreiber et al., 2008)
	Arabidopsis seedlings	96	42,000	Microsource Spectrum, Sigma TimTec Myia, ChemBridge NovaCore, ChemBridge DIVERSet	4-20 µM	CaBP22p::GUS reporter gene expression	(Knoth et al., 2009)
	Arabidopsis seedlings	96	6,800	Analyticon Discovery	10 µM	DEXp::evrRpm1-HA, EDp::avrRpm1 RPM1-MYC-induced effector triggered cell death	(Serrano et al., 2010)
	Arabidopsis seedlings	96	80	TimTec NP280	25 µM	Lesion-like spot development	(Schreiber et al., 2011)
	Arabidopsis cell culture	96	1,920	Microsource Spectrum	50 µM	Immune-related cell death	(Noutoshi et al., 2012a)*
	Arabidopsis cell culture	96	10,000	ChemBridge DIVERSet NovaCore NQ612	25 µg/mL	Immune-related cell death	(Noutoshi et al., 2012b)*
	Arabidopsis seedlings	96	6,800	Analyticon Discovery	10 µM	Anthocyanin accumulation	(Serrano et al., 2012)
Seed germination	Arabidopsis seeds	96	3,280	Sigma LOPAC, Microsource Spectrum	25 µM	Inhibition of germination	(Bassel et al., 2008;Park et al., 2009)
Accession specific hypocotyl elongation	Arabidopsis seedlings (various accessions)	96	13,280	ChemBridge DIVERSet, Sigma LOPAC, Microsource Spectrum	25 µM	Etiolated hypocotyl length	(Zhao et al., 2007)
Shoot regeneration	Arabidopsis seedlings	96	10,000	ChemBridge	10 µM	GAL4-GFP enhancer trap	(Motte et al., 2013)
Root development	Arabidopsis seedlings (pCYCB1;1::GUS)	96	10,000	ChemBridge DIVERSet	50 µM	Changes in *CYCB1;1* expression pattern in root tissue	(De Rybel et al., 2012)
	Arabidopsis seedlings	96	1,656	LATCA library + 80 selected compounds	8.3 µM	GFP enhancer trap line J2301 reporter gene expression and root growth	(Forde et al., 2013)
Growth and development	Arabidopsis seedlings	24	6,500	Korea Chemical Bank	2 µM	Plant morphology, growth rate, leaf color, flowering time and senescence	(Kim et al., 2010)
Gravitropism	Arabidopsis seedlings	24	10,000	ChemBridge DIVERSet	50-100 µM	Gravistimulated bending	(Surpin et al., 2005)
	Arabidopsis seedlings	24	10,000	ChemBridge DIVERSet	50-100 µM	Root length	(Christian et al., 2008)
Endomembrane trafficking	*Saccharomyces cerevisiae*	96	4,800	ChemBridge DIVERSet	10 µg/mL	Dot blot, anti-CPY antibody	(Zouhar et al., 2004)
	Tobacco pollen tube	96	2,016	Microsource Spectrum	50-100 µM	Pollen germination, pollen tube morphology	(Robert et al., 2008)
	Arabidopsis pollen tube	96	46,418	ChemBridge DIVERSet, ChemBridge NovaCore, Sigma TimTec Myria, LATCA library, CLICKables library	50-100 µM	Germination and growth of pollen tube	(Drakakaki et al., 2011)
	Arabidopsis seedlings (GFP-CESA3)	24	360	Selected chemicals from (Drakakaki et al., 2011)	15 µM	GFP-CESA3 localization	(Worden et al., 2015)

Targeted process	System	Plate size	Library size	Library source	Final conc.	Readout	Reference*
Peroxisome protein import	Arabidopsis (35Sp::GFP-MFP2) seedlings	24	70	ChemBridge DIVERSet Selected from (Surpin et al., 2005)	25 µM	35Sp::GFP-MFP2 reporter gene expression and peroxisome morphology and distribution	(Brown et al., 2011)
Cell morphology	Arabidopsis seedlings	96	20,000	ChemBridge DIVERSet	20-50 µM	Root or hypocotyl swelling	(DeBolt et al., 2007)
Cell expansion and cell morphogenesis	Tobacco BY-2 cell culture	96	4,080	Microsource Spectrum, LATCA library	25 µM	GFP-reporter gene expression and visualization of microtubules	(Yoneda et al., 2007)
Cell wall biosynthesis	*Pisum sativum* Golgi membranes	96	4,800	ChemBridge	N.d.	Radioactivity from radiolabeled-UDP-glucose	(Zabotina et al., 2008)*
Xylem differentiation	Arabidopsis (*acl5-1*) seedlings	24	1,680	Microsource Spectrum	10 µM	Microscopic xylem vessel differentiation	(Yoshimoto et al., 2012)

References

Armstrong, J.I., Yuan, S., Dale, J.M., Tanner, V.N., and Theologis, A. (2004). Identification of inhibitors of auxin transcriptional activation by means of chemical genetics in Arabidopsis. *Proc Natl Acad Sci U S A* 101**,** 14978-14983. doi: 10.1073/pnas.0404312101.

Bassel, G.W., Fung, P., Chow, T.F., Foong, J.A., Provart, N.J., and Cutler, S.R. (2008). Elucidating the germination transcriptional program using small molecules. *Plant Physiol* 147**,** 143-155. doi: 10.1104/pp.107.110841.

Brown, L.A., O'leary-Steele, C., Brookes, P., Armitage, L., Kepinski, S., Warriner, S.L., and Baker, A. (2011). A small molecule with differential effects on the PTS1 and PTS2 peroxisome matrix import pathways. *Plant J* 65**,** 980-990. doi: 10.1111/j.1365-313X.2010.04473.x.

Christian, M., Hannah, W.B., Luthen, H., and Jones, A.M. (2008). Identification of auxins by a chemical genomics approach. *J Exp Bot* 59**,** 2757-2767. doi: 10.1093/jxb/ern133.

De Rybel, B., Audenaert, D., Vert, G., Rozhon, W., Mayerhofer, J., Peelman, F., Coutuer, S., Denayer, T., Jansen, L., Nguyen, L., Vanhoutte, I., Beemster, G.T., Vleminckx, K., Jonak, C., Chory, J., Inzé, D., Russinova, E., and Beeckman, T. (2009). Chemical inhibition of a subset of Arabidopsis thaliana GSK3-like kinases activates brassinosteroid signaling. *Chem Biol* 16**,** 594-604. doi: 10.1016/j.chembiol.2009.04.008.

De Rybel, B., Audenaert, D., Xuan, W., Overvoorde, P., Strader, L.C., Kepinski, S., Hoye, R., Brisbois, R., Parizot, B., Vanneste, S., Liu, X., Gilday, A., Graham, I.A., Nguyen, L., Jansen, L., Njo, M.F., Inzé, D., Bartel, B., and Beeckman, T. (2012). A role for the root cap in root branching revealed by the non-auxin probe naxillin. *Nat Chem Biol* 8**,** 798-805. doi: 10.1038/nchembio.1044.

Debolt, S., Gutierrez, R., Ehrhardt, D.W., Melo, C.V., Ross, L., Cutler, S.R., Somerville, C., and Bonetta, D. (2007). Morlin, an inhibitor of cortical microtubule dynamics and cellulose synthase movement. *Proc Natl Acad Sci U S A* 104**,** 5854-5859. doi: 10.1073/pnas.0700789104.

Drakakaki, G., Robert, S., Szatmari, A.M., Brown, M.Q., Nagawa, S., Van Damme, D., Leonard, M., Yang, Z., Girke, T., Schmid, S.L., Russinova, E., Friml, J., Raikhel, N.V., and Hicks, G.R. (2011). Clusters of bioactive compounds target dynamic endomembrane networks in vivo. *Proc Natl Acad Sci U S A* 108**,** 17850-17855. doi: 10.1073/pnas.1108581108.

Forde, B.G., Cutler, S.R., Zaman, N., and Krysan, P.J. (2013). Glutamate signalling via a MEKK1 kinase-dependent pathway induces changes in Arabidopsis root architecture. *Plant J* 75**,** 1-10. doi: 10.1111/tpj.12201.

Gendron, J.M., Haque, A., Gendron, N., Chang, T., Asami, T., and Wang, Z.Y. (2008). Chemical genetic dissection of brassinosteroid-ethylene interaction. *Mol Plant* 1**,** 368-379. doi: 10.1093/mp/ssn005.

Hayashi, K., Ogino, K., Oono, Y., Uchimiya, H., and Nozaki, H. (2001). Yokonolide A, a new inhibitor of auxin signal transduction, from Streptomyces diastatochromogenes B59. *J Antibiot (Tokyo)* 54**,** 573-581.

He, W., Brumos, J., Li, H., Ji, Y., Ke, M., Gong, X., Zeng, Q., Li, W., Zhang, X., An, F., Wen, X., Li, P., Chu, J., Sun, X., Yan, C., Yan, N., Xie, D.Y., Raikhel, N., Yang, Z., Stepanova, A.N., Alonso, J.M., and Guo, H. (2011). A small-molecule screen identifies L-kynurenine as a competitive inhibitor of TAA1/TAR activity in ethylene-directed auxin biosynthesis and root growth in Arabidopsis. *Plant Cell* 23**,** 3944-3960. doi: 10.1105/tpc.111.089029.

Hu, Y., Callebert, P., Vandemoortel, I., Nguyen, L., Audenaert, D., Verschraegen, L., Vandenbussche, F., and Van Der Straeten, D. (2014). TR-DB: an open-access database of compounds affecting the ethylene-induced triple response in Arabidopsis. *Plant Physiol Biochem* 75**,** 128-137. doi: 10.1016/j.plaphy.2013.12.008.

Kim, J.Y., Henrichs, S., Bailly, A., Vincenzetti, V., Sovero, V., Mancuso, S., Pollmann, S., Kim, D., Geisler, M., and Nam, H.G. (2010). Identification of an ABCB/P-glycoprotein-specific inhibitor of auxin transport by chemical genomics. *J Biol Chem* 285**,** 23309-23317. doi: 10.1074/jbc.M110.105981.

Kim, T.H., Hauser, F., Ha, T., Xue, S., Böhmer, M., Nishimura, N., Munemasa, S., Hubbard, K., Peine, N., Lee, B.H., Lee, S., Robert, N., Parker, J.E., and Schroeder, J.I. (2011). Chemical genetics reveals negative regulation of abscisic acid signaling by a plant immune response pathway. *Curr Biol* 21**,** 990-997. doi: 10.1016/j.cub.2011.04.045.

Knoth, C., Salus, M.S., Girke, T., and Eulgem, T. (2009). The synthetic elicitor 3,5-dichloroanthranilic acid induces NPR1-dependent and NPR1-independent mechanisms of disease resistance in Arabidopsis. *Plant Physiol* 150**,** 333-347. doi: 10.1104/pp.108.133678.

Lin, L.C., Hsu, J.H., and Wang, L.C. (2010). Identification of novel inhibitors of 1-aminocyclopropane-1-carboxylic acid synthase by chemical screening in Arabidopsis thaliana. *J Biol Chem* 285**,** 33445-33456. doi: 10.1074/jbc.M110.132498.

Meesters, C., Mönig, T., Oeljeklaus, J., Krahn, D., Westfall, C.S., Hause, B., Jez, J.M., Kaiser, M., and Kombrink, E. (2014). A chemical inhibitor of jasmonate signaling targets JAR1 in Arabidopsis thaliana. *Nat Chem Biol* 10**,** 830-836. doi: 10.1038/nchembio.1591.

Min, Y.K., Asami, T., Fujioka, S., Murofushi, N., Yamaguchi, I., and Yoshida, S. (1999). New lead compounds for brassinosteroid biosynthesis inhibitors. *Bioorg Med Chem Lett* 9**,** 425-430.

Motte, H., Galuszka, P., Spichal, L., Tarkowski, P., Plihal, O., Smehilova, M., Jaworek, P., Vereecke, D., Werbrouck, S., and Geelen, D. (2013). Phenyl-adenine, identified in a LIGHT-DEPENDENT SHORT HYPOCOTYLS4-assisted chemical screen, is a potent compound for shoot regeneration through the inhibition of CYTOKININ OXIDASE/DEHYDROGENASE activity. *Plant Physiol* 161**,** 1229-1241. doi: 10.1104/pp.112.210716.

Nishimura, T., Hayashi, K., Suzuki, H., Gyohda, A., Takaoka, C., Sakaguchi, Y., Matsumoto, S., Kasahara, H., Sakai, T., Kato, J., Kamiya, Y., and Koshiba, T. (2014). Yucasin is a potent inhibitor of YUCCA, a key enzyme in auxin biosynthesis. *Plant J* 77**,** 352-366. doi: 10.1111/tpj.12399.

Nishimura, T., Matano, N., Morishima, T., Kakinuma, C., Hayashi, K., Komano, T., Kubo, M., Hasebe, M., Kasahara, H., Kamiya, Y., and Koshiba, T. (2012). Identification of IAA transport inhibitors including compounds affecting cellular PIN trafficking by two chemical screening approaches using maize coleoptile systems. *Plant Cell Physiol* 53**,** 1671-1682. doi: 10.1093/pcp/pcs112.

Noutoshi, Y., Ikeda, M., and Shirasu, K. (2012a). Diuretics prime plant immunity in Arabidopsis thaliana. *PLoS One* 7**,** e48443. doi: 10.1371/journal.pone.0048443.

Noutoshi, Y., Okazaki, M., Kida, T., Nishina, Y., Morishita, Y., Ogawa, T., Suzuki, H., Shibata, D., Jikumaru, Y., Hanada, A., Kamiya, Y., and Shirasu, K. (2012b). Novel plant immune-priming compounds identified via high-throughput chemical screening target salicylic acid glucosyltransferases in Arabidopsis. *Plant Cell* 24**,** 3795-3804. doi: 10.1105/tpc.112.098343.

Park, S.Y., Fung, P., Nishimura, N., Jensen, D.R., Fujii, H., Zhao, Y., Lumba, S., Santiago, J., Rodrigues, A., Chow, T.F., Alfred, S.E., Bonetta, D., Finkelstein, R., Provart, N.J., Desveaux, D., Rodriguez, P.L., Mccourt, P., Zhu, J.K., Schroeder, J.I., Volkman, B.F., and Cutler, S.R. (2009).

Abscisic acid inhibits type 2C protein phosphatases via the PYR/PYL family of START proteins. *Science* 324**,** 1068-1071. doi: 10.1126/science.1173041.

Robert, S., Chary, S.N., Drakakaki, G., Li, S., Yang, Z., Raikhel, N.V., and Hicks, G.R. (2008). Endosidin1 defines a compartment involved in endocytosis of the brassinosteroid receptor BRI1 and the auxin transporters PIN2 and AUX1. *Proc Natl Acad Sci U S A* 105**,** 8464-8469. doi: 10.1073/pnas.0711650105.

Savaldi-Goldstein, S., Baiga, T.J., Pojer, F., Dabi, T., Butterfield, C., Parry, G., Santner, A., Dharmasiri, N., Tao, Y., Estelle, M., Noel, J.P., and Chory, J. (2008). New auxin analogs with growth-promoting effects in intact plants reveal a chemical strategy to improve hormone delivery. *Proc Natl Acad Sci U S A* 105**,** 15190-15195. doi: 10.1073/pnas.0806324105.

Schreiber, K., Ckurshumova, W., Peek, J., and Desveaux, D. (2008). A high-throughput chemical screen for resistance to Pseudomonas syringae in Arabidopsis. *Plant J* 54**,** 522-531. doi: 10.1111/j.1365-313X.2008.03425.x.

Schreiber, K.J., Nasmith, C.G., Allard, G., Singh, J., Subramaniam, R., and Desveaux, D. (2011). Found in translation: high-throughput chemical screening in Arabidopsis thaliana identifies small molecules that reduce Fusarium head blight disease in wheat. *Mol Plant Microbe Interact* 24**,** 640-648. doi: 10.1094/MPMI-09-10-0210.

Serrano, M., Hubert, D.A., Dangl, J.L., Schulze-Lefert, P., and Kombrink, E. (2010). A chemical screen for suppressors of the avrRpm1-RPM1-dependent hypersensitive cell death response in Arabidopsis thaliana. *Planta* 231**,** 1013-1023. doi: 10.1007/s00425-010-1105-1.

Serrano, M., Kanehara, K., Torres, M., Yamada, K., Tintor, N., Kombrink, E., Schulze-Lefert, P., and Saijo, Y. (2012). Repression of sucrose/ultraviolet B light-induced flavonoid accumulation in microbe-associated molecular pattern-triggered immunity in Arabidopsis. *Plant Physiol* 158**,** 408-422. doi: 10.1104/pp.111.183459.

Serrano, M., Robatzek, S., Torres, M., Kombrink, E., Somssich, I.E., Robinson, M., and Schulze-Lefert, P. (2007). Chemical interference of pathogen-associated molecular pattern-triggered immune responses in Arabidopsis reveals a potential role for fatty-acid synthase type II complex-derived lipid signals. *J Biol Chem* 282**,** 6803-6811. doi: 10.1074/jbc.M608792200.

Spring, D.R., Krishnan, S., Blackwell, H.E., and Schreiber, S.L. (2002). Diversity-Oriented Synthesis of Biaryl-Containing Medium Rings Using a One Bead/One Stock Solution Platform. *Journal of the American Chemical Society* 124**,** 1354-1363. doi: 10.1021/ja017248o.

Surpin, M., Rojas-Pierce, M., Carter, C., Hicks, G.R., Vasquez, J., and Raikhel, N.V. (2005). The power of chemical genomics to study the link between endomembrane system components and the gravitropic response. *Proc Natl Acad Sci U S A* 102**,** 4902-4907. doi: 10.1073/pnas.0500222102.

Toth, R., Gerding-Reimers, C., Deeks, M.J., Menninger, S., Gallegos, R.M., Tonaco, I.A., Hubel, K., Hussey, P.J., Waldmann, H., and Coupland, G. (2012). Prieurianin/endosidin 1 is an actin-stabilizing small molecule identified from a chemical genetic screen for circadian clock effectors in Arabidopsis thaliana. *Plant J* 71**,** 338-352. doi: 10.1111/j.1365-313X.2012.04991.x.

Tsuchiya, Y., Vidaurre, D., Toh, S., Hanada, A., Nambara, E., Kamiya, Y., Yamaguchi, S., and Mccourt, P. (2010). A small-molecule screen identifies new functions for the plant hormone strigolactone. *Nat Chem Biol* 6**,** 741-749. doi: 10.1038/nchembio.435.

Worden, N., Wilkop, T.E., Esteve, V.E., Jeannotte, R., Lathe, R., Vernhettes, S., Weimer, B., Hicks, G., Alonso, J., Labavitch, J., Persson, S., Ehrhardt, D., and Drakakaki, G. (2015). CESA TRAFFICKING INHIBITOR Inhibits cellulose deposition and interferes with the trafficking of cellulose synthase complexes and their associated proteins KORRIGAN1 and POM2/CELLULOSE SYNTHASE INTERACTIVE PROTEIN1. *Plant Physiol* 167**,** 381-393. doi: 10.1104/pp.114.249003.

Yoneda, A., Higaki, T., Kutsuna, N., Kondo, Y., Osada, H., Hasezawa, S., and Matsui, M. (2007). Chemical genetic screening identifies a novel inhibitor of parallel alignment of cortical microtubules and cellulose microfibrils. *Plant Cell Physiol* 48**,** 1393-1403. doi: 10.1093/pcp/pcm120.

Yoshimoto, K., Noutoshi, Y., Hayashi, K., Shirasu, K., Takahashi, T., and Motose, H. (2012). A chemical biology approach reveals an opposite action between thermospermine and auxin in xylem development in Arabidopsis thaliana. *Plant Cell Physiol* 53**,** 635-645. doi: 10.1093/pcp/pcs017.

Yoshitani, N., Satou, K., Saito, K., Suzuki, S., Hatanaka, H., Seki, M., Shinozaki, K., Hirota, H., and Yokoyama, S. (2005). A structure-based strategy for discovery of small ligands binding to functionally unknown proteins: combination of in silico screening and surface plasmon resonance measurements. *Proteomics* 5**,** 1472-1480. doi: 10.1002/pmic.200401032.

Zabotina, O., Malm, E., Drakakaki, G., Bulone, V., and Raikhel, N. (2008). Identification and preliminary characterization of a new chemical affecting glucosyltransferase activities involved in plant cell wall biosynthesis. *Mol Plant* 1**,** 977-989. doi: 10.1093/mp/ssn055.

Zhao, Y., Chow, T.F., Puckrin, R.S., Alfred, S.E., Korir, A.K., Larive, C.K., and Cutler, S.R. (2007). Chemical genetic interrogation of natural variation uncovers a molecule that is glycoactivated. *Nat Chem Biol* 3**,** 716-721. doi: 10.1038/nchembio.2007.32.

Zouhar, J., Hicks, G.R., and Raikhel, N.V. (2004). Sorting inhibitors (Sortins): Chemical compounds to study vacuolar sorting in Arabidopsis. *Proc Natl Acad Sci U S A* 101**,** 9497-9501. doi: 10.1073/pnas.0402121101.
